# Diabetic Ketoacidosis-Induced Cardiomyopathy and Reversible Dialysis-Dependent Renal Failure With Successful Outcome: A Report of a Rare Case

**DOI:** 10.7759/cureus.31711

**Published:** 2022-11-20

**Authors:** Ahmed Abbas, Nirav Patel, Riyashat Kazmi, Noreen Mirza, Richard Miller, Joaquim Correia

**Affiliations:** 1 Internal Medicine, Saint Michael's Medical Center, Newark, USA; 2 Pulmonary and Critical Care Medicine, Saint Michael's Medical Center, Newark, USA; 3 Cardiology and Electrophysiology, Saint Michael's Medical Center, Newark, USA

**Keywords:** hypertriglyceridemia, dialysis, rhabdomyolysis, stress cardiomyopathy, cardiogenic shock, cardiomyopathy, takotsubo, hyperglycemia, dka

## Abstract

Stress-induced or Takotsubo cardiomyopathy (TCM) is a phenomenon that typically occurs in postmenopausal women in the setting of acute emotional or medical stressors. It typically causes reversible akinesis of the heart apex with opposite hyperdynamic basal segments. An electrocardiogram (ECG) would show ischemic ST elevation in anterior leads in >90% of cases with elevated troponin, yet coronary angiography rules out occlusive disease. Takotsubo cardiomyopathy in the setting of diabetic ketoacidosis (DKA) is a rare phenomenon that has been attributed to severe acidosis. Here, we report the case of a 37-year-old male with severe DKA that was complicated by stress cardiomyopathy and progressed to cardiogenic shock.

## Introduction

Stress-induced or Takotsubo cardiomyopathy (TCM) is a syndrome of transient regional myocardial systolic dysfunction which is usually apical or midventricular that extends beyond the territory of a single coronary artery, and in the absence of angiographic documentation of coronary artery disease. The incidence of such a phenomenon remains unknown, but it is more described in women with a mean age of 66 years with no prior history of coronary artery disease in the setting of an acute psychiatric or catastrophic medical condition [1].

There are several reported cases of TCM in the setting of diabetic ketoacidosis (DKA) in the literature. Most of which were linked to underlying sepsis, thyrotoxicosis, hypothermia, or simply the systemic inflammatory response [2-6]. To our knowledge, there have been few cases of non-TCM in the setting of DKA. We report a case of DKA-induced global cardiomyopathy that did not follow the Takotsubo pattern with successful management, to shed some light on the risk profile for such a complication, and to emphasize the importance of early recognition and early employment of vasopressors to avoid fluid overload. 

## Case presentation

A 37-year-old Hispanic man with a past medical history of poorly controlled type 2 diabetes mellitus (T2DM) diagnosed 10 months prior to presentation, was brought to the emergency room after being found unconscious in bed with yellow vomitus. According to his wife, he was non-compliant with his insulin regimen while craving sweets and had lost 30 lbs of weight unintentionally over the past two months. On arrival, the patient was only responding to sternal rub. On physical examination, he had a blood pressure of 70/40 mmHg, heart rate of 110 beats per minute, dry mouth with dark blood around, a Glasgow-coma scale (GCS) of 8, and tender left upper abdomen. The initial laboratory workup is shown in Table 1.

**Table 1:** Initial laboratory data pH: Potential of hydrogen

Laboratory parameter	Value	Reference range
Random plasma glucose	2088	70-140 (mg/dl)
Glycated hemoglobin (A1C)	>14	4-5.6 (%)
Beta-hydroxybutyrate	3	0.02-0.27 (mmol/l)
pH	7.25	7.35-7.45
Bicarbonate	13	22-28 (mmol/l)
Serum anion gap	29 (delta ratio 1.5)	5-15 (mmol/l)
Lactic acid	9	0-2 (mmol/l)
Blood urea nitrogen	72	6-24 (mg/dl)
Creatinine	7.6 (unknown baseline)	0.6-1.2 (mg/dl)
Phosphorus	0.9	2.1-4.3 (mg/dl)
Creatine kinase	2200	52-336 (U/l)
Lipase	2900	73-393 (U/l)
Triglycerides	770	0-150 (mg/dl)
High sensitivity troponin	18 (peaked at 505)	0-76 (ng/l)

**Figure 1:**
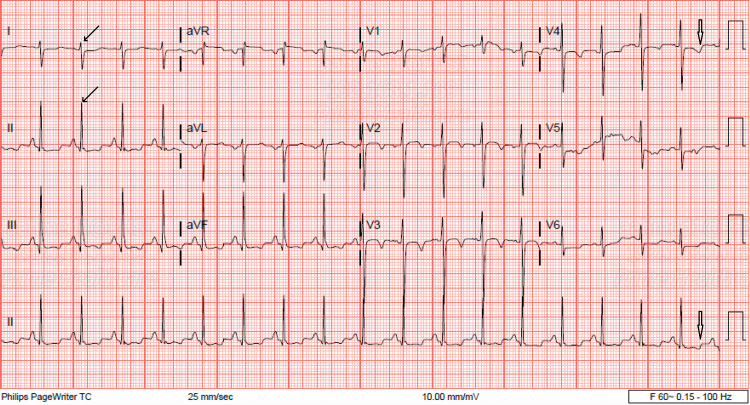
ECG showing sinus tachycardia with right axis deviation (solid arrows) and repolarization abnormalities (hollow arrows).

The electrocardiogram (ECG) demonstrated sinus tachycardia with right axis deviation and repolarization abnormalities but no record of ST-segment elevation as in Figure 1.

**Figure 2:**
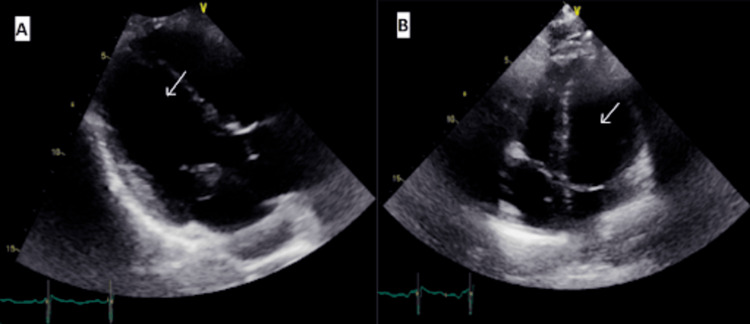
Initial transthoracic echo Parasternal long axis (A) and four-chamber (B) views show diffuse hypokinesis of the left ventricle (white arrow) with an ejection fraction of less than 30%.

The patient had increasing troponins for which the cardiologist advised they were more likely to demand ischemia in the setting of hypotension and DKA. For the workup of hypotension, initial echocardiography showed diffuse global hypokinesis with a left ventricle ejection fraction (LVEF) of 30% as in Figure 2. 

The patient was intubated in the field to secure his airway, and soon after dark brown emesis was noted. Abdominal computed tomography (CT) in view of elevated lipase had no pancreatitis. He received multiple fluid boluses with persistent hypotension and was started on DKA protocol, empiric antibiotics, and norepinephrine, which was later intensified with a seven-day course of vasopressin and dobutamine for a target central venous oxygen saturation (SVO2) > 70%. 

With initial aggressive fluid resuscitation in the setting of poor renal functions, the patient developed pulmonary, lower limb, and scrotal edema. Given his poor urine output and ongoing rhabdomyolysis, he received 12 days of hemodialysis. Septic workup including chest X-ray, urinalysis, and blood cultures were all negative, hence antibiotics were discontinued. A urine drug screening was negative for cocaine as a culprit for cardiomyopathy, a 24-hour electroencephalogram (EEG) ruled out non-convulsive status epilepticus, and an MRI brain was negative for masses or infarcts. 

**Figure 3:**
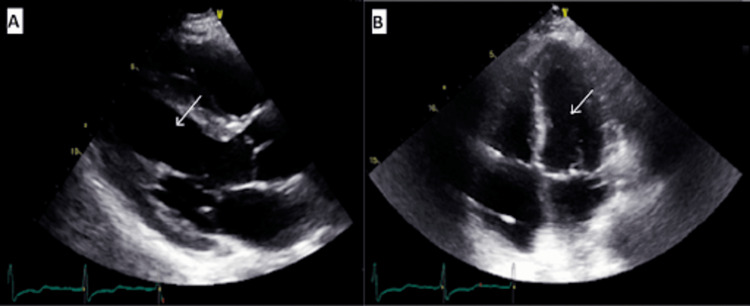
Repeat transthoracic echo two weeks after presentation Parasternal long axis (A) and four-chamber (B) views show a left ventricle (white arrow) ejection fraction of 60% with normal wall motion.

A repeat echocardiography two weeks later showed an improved LVEF of 60% with normal wall motion as in Figure 3.

Hence the patient was successfully weaned off pressors and extubated. His renal failure resolved, the dialysis catheter was subsequently removed and he was downgraded to telemetry. Endocrinology recommended discharge on insulin (70/30) with 18 units before breakfast and 10 units at bedtime with outpatient follow-up and diabetes education. The patient had a total hospital stay of 23 days.

## Discussion

Diabetic ketoacidosis is the most common hyperglycemic emergency in people with diabetes mellitus. It results from absolute or relative insulin deficiency with a concomitant increase of counter-regulatory stress hormones; hence it occurs twice as common in type 1 compared to type 2 DM [7]. The most common DKA complication is treatment-induced hypokalemia and hypoglycemia which is generally mild and reversible with careful basic metabolic panel (BMP) monitoring [8,9]. Acute kidney injury occurs in up to 50% of adults with DKA, usually stage 1 pre-renal, and correlates with the degree of hyperglycemia. A full-blown renal failure requiring dialysis is less common and is associated with rhabdomyolysis. Rhabdomyolysis in turn strongly correlates with severe hypophosphatemia as in our case [7].

Stress-induced or Takotsubo cardiomyopathy (TCM) is a syndrome of transient regional myocardial systolic dysfunction which is usually apical or midventricular that extends beyond the territory of a single coronary artery in absence of angiographic documentation of coronary artery disease. The incidence of such a phenomenon remains unknown, but it’s more described in women of mean age 66 years with no prior history of coronary artery disease in the setting of acute psychiatric or catastrophic medical condition [1].

According to the International Takotsubo Registry study of 1759 patients, the most common presenting symptom was chest pain at 75.9%, and the most common ECG abnormality was ST-segment elevation in the anterior precordial leads at 43.7% [10]. The four diagnostic criteria of TCM proposed by the Mayo clinic are: transient left ventricular (LV) systolic dysfunction, absent obstructive coronary artery disease on angiography, new ECG abnormalities or elevated troponins, and absent pheochromocytoma or myocarditis [11].

Proposed mechanisms of elevated cardiac biomarkers and cardiomyopathy in the setting of DKA include: severe acidemia increases intracellular Calcium with proteolysis and release of troponin; high level of free fatty acids incorporate into the lipid cell membrane with the formation of micelle and rupture; increased counter-regulatory stress hormones as adrenaline increases the myocardial oxygen demand resulting in myonecrosis [12]; pro-inflammatory cytokines associated with systemic inflammatory response syndrome (SIRS) increase free radicals that inhibit the contractile proteins causing myocardial stunning [2].

## Conclusions

Diabetic ketoacidosis-induced cardiomyopathy whether Takotsubo or non-Takotsubo is an increasingly recognized complication that needs early recognition and early workup to exclude reversible causes most notorious of which is an acute coronary syndrome. Counseling on diabetes control and an interdisciplinary team approach with a primary care physician, endocrinologist, cardiologist, and nephrologist remain the most cost-effective means to prevent DKA with its associated morbidity and mortality. Our case had a miraculous recovery despite his initial sequential organ failure assessment (SOFA) score of 14 points representing >95% mortality given his relatively young age and few comorbidities which unfortunately is not the case in other scenarios. Also, this case proves a great deal needs to be done by policymakers to address health care disparities and lesser diabetes care among marginalized populations such as African Americans and Hispanics like our patient.
